# Amino­guanidinium hydrogen fumarate

**DOI:** 10.1107/S1600536809004553

**Published:** 2009-02-18

**Authors:** Swaminathan Murugavel, Gnanavelu Ganesh, Arunachalathevar Subbiah Pandi, Subbiah Govindarajan, Rajendran Selvakumar

**Affiliations:** a Department of Physics, Thanthai Periyar Government Institute of Technology, Vellore 632 002, India; b Department of Physics, SMK Fomra Institute of Technology, Thaiyur, Chennai 603 103, India; c Department of Physics, Presidency College (Autonomous), Chennai 600 005, India; d Department of Chemistry, Bharathiar University, Coimbatore 641 046, India

## Abstract

The title compound, CH_7_N_4_^+^·C_4_H_3_O_4_^−^, is a molecular salt in which the amino­guanidinium cations and fumarate monoanions are close to planar, with maximum deviations of 0.011 (1) and 0.177 (1) Å, respectively. The crystal packing is stabilized by inter­molecular N—H⋯O and O—H⋯O hydrogen bonds.

## Related literature

For related structures, see: Adams (1977 ▶); Akella & Keszler (1994 ▶); Mullen & Hellner (1978 ▶). For biological applications, see: Makita *et al.* (1995 ▶); Brownlee *et al.* (1986 ▶).
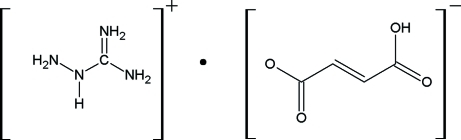


## Experimental

### 

#### Crystal data

CH_7_N_4_^+^·C_4_H_3_O_4_^−^
*M* *_r_* = 190.17
Monoclinic, 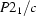

*a* = 6.3869 (3) Å
*b* = 19.8731 (10) Å
*c* = 7.0482 (4) Å
β = 114.688 (3)°
*V* = 812.84 (8) Å^3^
*Z* = 4
Mo *K*α radiation
μ = 0.13 mm^−1^
*T* = 293 K
0.26 × 0.15 × 0.15 mm

#### Data collection

Bruker APEXII CCD area-detector diffractometer
Absorption correction: multi-scan (*SADABS*; Sheldrick, 1996 ▶) *T*_min_ = 0.966, *T*_max_ = 0.976
10713 measured reflections
2340 independent reflections
1824 reflections with *I* > 2σ(*I*)
*R*_int_ = 0.028

#### Refinement

*R*[*F* ^2^ > 2σ(*F* ^2^)] = 0.040
*wR*(*F* ^2^) = 0.132
*S* = 1.04
2340 reflections
146 parameters
H atoms treated by a mixture of independent and constrained refinement
Δρ_max_ = 0.33 e Å^−3^
Δρ_min_ = −0.31 e Å^−3^

### 

Data collection: *APEX2* (Bruker, 2004 ▶); cell refinement: *APEX2*; data reduction: *SAINT* (Bruker, 2004 ▶); program(s) used to solve structure: *SHELXS97* (Sheldrick, 2008 ▶); program(s) used to refine structure: *SHELXL97* (Sheldrick, 2008 ▶); molecular graphics: *ORTEP-3* (Farrugia, 1997 ▶); software used to prepare material for publication: *SHELXL97* and *PLATON* (Spek, 2009 ▶).

## Supplementary Material

Crystal structure: contains datablocks global, I. DOI: 10.1107/S1600536809004553/wm2220sup1.cif

Structure factors: contains datablocks I. DOI: 10.1107/S1600536809004553/wm2220Isup2.hkl

Additional supplementary materials:  crystallographic information; 3D view; checkCIF report

## Figures and Tables

**Table 1:** Hydrogen-bond geometry (Å, °)

*D*—H⋯*A*	*D*—H	H⋯*A*	*D*⋯*A*	*D*—H⋯*A*
O8—H8⋯O7^i^	0.82	1.68	2.489 (1)	168
N10—H10*A*⋯O7^ii^	0.91 (2)	2.09 (2)	2.993 (1)	177 (2)
N11—H11*A*⋯O6^ii^	0.92 (2)	1.91 (2)	2.827 (2)	173 (2)

Symmetry codes: (i) 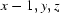; (ii) 
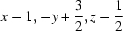
.

**Fig. 1.**
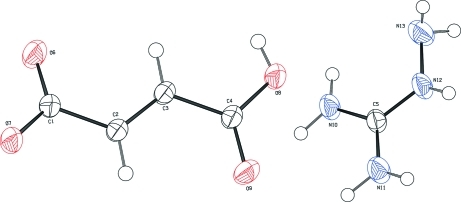
The molecular structure of the ions present in compound (I) showing 50% probability displacement ellipsoids.

**Fig. 2.**
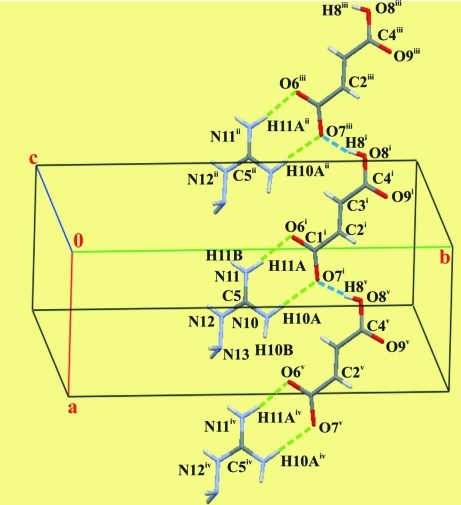
N—H···O and O—H···O hydrogen bonds (dotted lines) in the title compound. [Symmetry codes: (i) x-1, -y+3/2, z-1/2; (ii)x-1, y, z].

## supplementary crystallographic information

### Comment

Aminoguanadine is an early inhibitor of advanced glycosylation end products (Makita *et al.*, 1995). It helps prevent proteins cross-linking and is being used in diabetes, atherosclerosis, renal and aging disorders (Brownlee *et al.*, 1986). Aminoguanadine is a highly reactive nucleophillic reagent that reacts with many biological molecules (pyridoxal phosphate, pyruvate, glucose, malondialdehyde, and others). The crystal structures of several guanidinium salts have previously been reported over the last three decades (Adams, 1977; Mullen & Hellner, 1978). Here we report the crystal structure of the title compound, aminoguanidinium hydrogenfumarate, (I), (Fig. 1). In the molecular salt (I), the aminoguanidinium cation and fumarate anion each are nearly planar, with maximum deviations of -0.011 (1) Å and -0.177 (1) Å for atom N12 and O7, respectively ( Fig. 1). The bond lengths in (I) are comparable with the corresponding values observed in related structures (Akella & Keszler , 1994). The angle between the best planes of the aminoguanidinium cation and the fumarate anion is 12.78 (6)°. Atom N10 and N11 in the molecule at (*x*, *y*, *z*) donate one proton each to the atoms O7 and O6 in the molecule at (-*1+x*, *3/2-y*, -*1/2+z*), generating a *R*_2_^2^(8) ring motif (Table 1 and Fig. 2). Also, an O—H···O interaction is observed (Table 1). Thus, the symmetry-related molecules are cross linked by these hydrogen bonds to generate a three-dimensional network.

### Experimental

Needle-shaped single crystals of aminoguanidium hydrogenfumarate were prepared by slow evaporation of the aqueous solution obtained by dissolving of aminoguanidinium hydrogencarbonate (0.136g; 0.001mol) in fumaric acid (0.116 g; 1 mmol) solution (30 mL) at ambient condition. Colourless single crystals suitable for X-ray diffraction obtained after four days were collected, washed with ethanol and air dried.

### Refinement

All N bound H atoms were located in a difference map and refined freely. All other H atoms were fixed geometrically and allowed to ride on their parent atoms, with distances of O—H = 0.82Å and C—H = 0.93Å with *U*_iso_(H)= 1.2*U*_eq_.

### Crystal data

**Table:**

CH_7_N_4_^+^·C_4_H_3_O_4_^−^	*F*(000) = 400
*M**_r_* = 190.17	*D*_x_ = 1.554 Mg m^−^^3^
Monoclinic, *P*2_1_/*c*	Mo *K*α radiation, λ = 0.71073 Å
Hall symbol: -P 2ybc	Cell parameters from 1824 reflections
*a* = 6.3869 (3) Å	θ = 2–29.9°
*b* = 19.8731 (10) Å	µ = 0.13 mm^−^^1^
*c* = 7.0482 (4) Å	*T* = 293 K
β = 114.688 (3)°	Block, colourless
*V* = 812.84 (8) Å^3^	0.26 × 0.15 × 0.15 mm
*Z* = 4	

### Data collection

**Table:**

Bruker APEXII CCD area-detector diffractometer	2340 independent reflections
Radiation source: fine-focus sealed tube	1824 reflections with *I* > 2σ(*I*)
graphite	*R*_int_ = 0.028
Detector resolution: 10.0 pixels mm^-1^	θ_max_ = 29.9°, θ_min_ = 2.1°
ω scans	*h* = −8→7
Absorption correction: multi-scan (*SADABS*; Sheldrick, 1996)	*k* = −24→27
*T*_min_ = 0.966, *T*_max_ = 0.976	*l* = −9→9
10713 measured reflections	

### Refinement

**Table:**

Refinement on *F*^2^	Primary atom site location: structure-invariant direct methods
Least-squares matrix: full	Secondary atom site location: difference Fourier map
*R*[*F*^2^ > 2σ(*F*^2^)] = 0.040	Hydrogen site location: inferred from neighbouring sites
*wR*(*F*^2^) = 0.132	H atoms treated by a mixture of independent and constrained refinement
*S* = 1.04	*w* = 1/[σ^2^(*F*_o_^2^) + (0.0756*P*)^2^ + 0.1408*P*] where *P* = (*F*_o_^2^ + 2*F*_c_^2^)/3
2340 reflections	(Δ/σ)_max_ < 0.001
146 parameters	Δρ_max_ = 0.33 e Å^−^^3^
0 restraints	Δρ_min_ = −0.31 e Å^−^^3^

### Special details

**Table:**

Geometry. All esds (except the esd in the dihedral angle between two l.s. planes) are estimated using the full covariance matrix. The cell esds are taken into account individually in the estimation of esds in distances, angles and torsion angles; correlations between esds in cell parameters are only used when they are defined by crystal symmetry. An approximate (isotropic) treatment of cell esds is used for estimating esds involving l.s. planes.
Refinement. Refinement of F^2^ against ALL reflections. The weighted R-factor wR and goodness of fit S are based on F^2^, conventional R-factors R are based on F, with F set to zero for negative F^2^. The threshold expression of F^2^ > 2sigma(F^2^) is used only for calculating R-factors(gt) etc. and is not relevant to the choice of reflections for refinement. R-factors based on F^2^ are statistically about twice as large as those based on F, and R- factors based on ALL data will be even larger.

### Fractional atomic coordinates and isotropic or equivalent isotropic displacement parameters (Å^2^)

**Table:**

	*x*	*y*	*z*	*U*_iso_*/*U*_eq_	
H11B	0.243 (3)	0.4484 (12)	0.254 (3)	0.057 (5)*	
H12	0.573 (3)	0.3932 (11)	0.302 (3)	0.056 (5)*	
H13A	0.861 (4)	0.3941 (12)	0.204 (3)	0.072 (6)*	
C1	1.12336 (19)	0.84396 (5)	0.68336 (19)	0.0281 (3)	
C2	0.9984 (2)	0.78049 (5)	0.68320 (19)	0.0274 (2)	
H2	1.0738	0.7396	0.6953	0.033*	
C3	0.7863 (2)	0.78034 (6)	0.6666 (2)	0.0292 (3)	
H3	0.7125	0.8212	0.6595	0.035*	
C4	0.6603 (2)	0.71704 (5)	0.65888 (19)	0.0279 (3)	
O6	1.04931 (17)	0.89803 (5)	0.71551 (19)	0.0466 (3)	
O7	1.30209 (15)	0.83829 (4)	0.64654 (17)	0.0362 (2)	
O8	0.45191 (15)	0.72255 (4)	0.64769 (17)	0.0374 (2)	
H8	0.4189	0.7625	0.6465	0.056*	
O9	0.74557 (17)	0.66199 (4)	0.66416 (19)	0.0434 (3)	
H10A	0.521 (3)	0.5783 (9)	0.214 (3)	0.046 (5)*	
H10B	0.747 (4)	0.5370 (10)	0.245 (3)	0.057 (5)*	
H11A	0.221 (3)	0.5225 (10)	0.224 (3)	0.053 (5)*	
C5	0.5133 (2)	0.48405 (6)	0.25398 (18)	0.0277 (3)	
N10	0.6094 (2)	0.54110 (5)	0.23653 (19)	0.0350 (3)	
N11	0.3028 (2)	0.48299 (6)	0.2428 (2)	0.0412 (3)	
N12	0.6265 (2)	0.42625 (5)	0.2812 (2)	0.0361 (3)	
N13	0.8519 (2)	0.42556 (6)	0.2974 (2)	0.0431 (3)	
H13B	0.935 (4)	0.4125 (10)	0.424 (3)	0.061 (6)*	

### Atomic displacement parameters (Å^2^)

**Table:**

	*U*^11^	*U*^22^	*U*^33^	*U*^12^	*U*^13^	*U*^23^
C1	0.0233 (5)	0.0217 (5)	0.0413 (6)	−0.0015 (4)	0.0153 (5)	0.0003 (4)
C2	0.0253 (5)	0.0198 (5)	0.0400 (6)	−0.0004 (4)	0.0166 (5)	0.0010 (4)
C3	0.0270 (6)	0.0179 (5)	0.0477 (7)	−0.0010 (4)	0.0206 (5)	−0.0004 (4)
C4	0.0264 (5)	0.0200 (5)	0.0412 (6)	−0.0018 (4)	0.0180 (5)	0.0000 (4)
O6	0.0395 (5)	0.0226 (4)	0.0891 (8)	−0.0035 (4)	0.0382 (6)	−0.0077 (5)
O7	0.0291 (4)	0.0264 (4)	0.0620 (6)	−0.0027 (3)	0.0279 (4)	0.0003 (4)
O8	0.0280 (4)	0.0228 (4)	0.0683 (7)	−0.0027 (3)	0.0272 (4)	−0.0004 (4)
O9	0.0390 (5)	0.0196 (4)	0.0796 (7)	0.0023 (3)	0.0328 (5)	0.0019 (4)
C5	0.0301 (6)	0.0208 (5)	0.0345 (6)	0.0007 (4)	0.0158 (5)	0.0002 (4)
N10	0.0341 (6)	0.0206 (5)	0.0553 (7)	−0.0007 (4)	0.0238 (5)	0.0025 (4)
N11	0.0350 (6)	0.0254 (5)	0.0713 (9)	0.0000 (5)	0.0304 (6)	0.0024 (5)
N12	0.0353 (6)	0.0193 (5)	0.0599 (7)	0.0021 (4)	0.0260 (5)	0.0052 (4)
N13	0.0345 (6)	0.0348 (6)	0.0638 (9)	0.0076 (5)	0.0244 (6)	0.0025 (6)

### Geometric parameters (Å, °)

**Table:**

C1—O6	1.2325 (14)	C5—N10	1.3190 (15)
C1—O7	1.2770 (14)	C5—N12	1.3278 (15)
C1—C2	1.4922 (15)	N10—H10A	0.905 (18)
C2—C3	1.3105 (16)	N10—H10B	0.86 (2)
C2—H2	0.9300	N11—H11B	0.80 (2)
C3—C4	1.4820 (15)	N11—H11A	0.92 (2)
C3—H3	0.9300	N12—N13	1.3960 (16)
C4—O9	1.2159 (14)	N12—H12	0.78 (2)
C4—O8	1.3044 (14)	N13—H13A	0.93 (2)
O8—H8	0.8200	N13—H13B	0.87 (2)
C5—N11	1.3136 (17)		
			
O6—C1—O7	123.96 (10)	N11—C5—N12	118.46 (11)
O6—C1—C2	119.40 (10)	N10—C5—N12	120.71 (11)
O7—C1—C2	116.64 (10)	C5—N10—H10A	115.9 (12)
C3—C2—C1	122.32 (10)	C5—N10—H10B	114.5 (14)
C3—C2—H2	118.8	H10A—N10—H10B	129.6 (19)
C1—C2—H2	118.8	C5—N11—H11B	121.4 (15)
C2—C3—C4	122.04 (10)	C5—N11—H11A	120.0 (12)
C2—C3—H3	119.0	H11B—N11—H11A	119 (2)
C4—C3—H3	119.0	C5—N12—N13	120.06 (11)
O9—C4—O8	120.67 (10)	C5—N12—H12	120.1 (15)
O9—C4—C3	122.22 (10)	N13—N12—H12	119.5 (15)
O8—C4—C3	117.11 (10)	N12—N13—H13A	108.3 (15)
C4—O8—H8	109.5	N12—N13—H13B	104.9 (14)
N11—C5—N10	120.83 (11)	H13A—N13—H13B	109.7 (19)
			
O6—C1—C2—C3	16.50 (19)	C2—C3—C4—O8	178.28 (11)
O7—C1—C2—C3	−162.59 (12)	N11—C5—N12—N13	−178.90 (13)
C1—C2—C3—C4	177.74 (11)	N10—C5—N12—N13	1.8 (2)
C2—C3—C4—O9	−1.2 (2)		

### Hydrogen-bond geometry (Å, °)

**Table:**

*D*—H···*A*	*D*—H	H···*A*	*D*···*A*	*D*—H···*A*
O8—H8···O7^i^	0.82	1.68	2.489 (1)	168
N10—H10A···O7^ii^	0.91 (2)	2.09 (2)	2.993 (1)	177 (2)
N11—H11A···O6^ii^	0.92 (2)	1.91 (2)	2.827 (2)	173 (2)

Symmetry codes: (i) *x*−1, *y*, *z*; (ii) *x*−1, −*y*+3/2, *z*−1/2.
